# Zebrafish: unraveling genetic complexity through duplicated genes

**DOI:** 10.1007/s00427-024-00720-6

**Published:** 2024-07-30

**Authors:** Maliha Tasnim, Preston Wahlquist, Jonathon T. Hill

**Affiliations:** Department of Cell Biology and Physiology, Brigham Young University, 701 E. University Pkwy, Provo, UT 84602 USA

**Keywords:** Gene duplication, Neofunctionalization, Subfunctionalization, Non-functionalization, Zebrafish, Genetic complexity, Protein-encoding genes

## Abstract

The zebrafish is an invaluable model organism for genetic, developmental, and disease research. Although its high conservation with humans is often cited as justification for its use, the zebrafish harbors oft-ignored genetic characteristics that may provide unique insights into gene structure and function. Zebrafish, along with other teleost fish, underwent an additional round of whole genome duplication after their split from tetrapods—resulting in an abundance of duplicated genes when compared to other vertebrates. These duplicated genes have evolved in distinct ways over the ensuing 350 million years. Thus, each gene within a duplicated gene pair has nuanced differences that create a unique identity. By investigating both members of the gene pair together, we can elucidate the mechanisms that underly protein structure and function and drive the complex interplay within biological systems, such as signal transduction cascades, genetic regulatory networks, and evolution of tissue and organ function. It is crucial to leverage such studies to explore these molecular dynamics, which could have far-reaching implications for both basic science and therapeutic development. Here, we will review the role of gene duplications and the existing models for gene divergence and retention following these events. We will also highlight examples within each of these models where studies comparing duplicated genes in the zebrafish have yielded key insights into protein structure, function, and regulation.

## Introduction

Zebrafish are a valuable model for human disease studies due to the high degree of genetic conservation between the two species—approximately 70% of human protein-coding genes, including the majority of genes implicated in disease, have highly conserved orthologs in zebrafish (Adhish & Manjubala 2023; Barut & Zon 2000; Goldsmith & Jobin 2012; Howe et al. 2013; Santoriello & Zon 2012; Shehwana & Konu 2019). Genetic manipulation methods in the zebrafish, such as CRISPR mutagenesis (Hwang et al. 2013) and transgenic line generation (Streisinger et al. 1981), are also well-established and increasingly complemented by targeted gene insertion methods (Ata et al. 2018; Auer et al. 2014; Ranawakage et al. 2021). As a result, the zebrafish is one of the most commonly used model organisms and has been employed in studies on tissue and organ development, a wide range of human diseases, and drug discovery efforts (Chia et al. 2022; Choi et al. 2021; Patton et al. 2021; Teame et al. 2019). These studies have profoundly enhanced our knowledge of genetics, development, and disease, making zebrafish indispensable in biomedical research now and for the foreseeable future.

Despite the benefits of zebrafish’s genetic parallels with humans, the species’ greatest value as a model organism may come from a distinctive aspect of its genome—the remarkable abundance of duplicate genes within its genome. Most vertebrates share two rounds of genome duplication that occurred shortly after their split from invertebrates. These events, often referred to as the first (1R) and second (2R) rounds of genome duplication, are thought to have taken place approximately 500 to 600 million years ago, before the radiation of the extant vertebrate classes (Ohno 1970). In addition to the two vertebrate-wide genome duplication events, the teleost clade (which includes zebrafish) has undergone a third round of genome duplication known as the teleost-specific whole genome duplication (TS-3R WGD). This event is thought to have contributed to the considerable expansion and diversification of their genetic repertoire, resulting in an explosive evolutionary radiation that rapidly created the largest and most diverse vertebrate clade (Inoue et al. 2015; Postlethwait et al. 2000). This event is supported by multiple lines of evidence, including a detailed study of genomic architecture that showed that 3440 gene pairs—26% of the genes analyzed—exist within double-conserved synteny (DCS) blocks (Howe et al. 2013). In zebrafish, these duplications are supplemented by an apparent propensity for gene duplication. One study determined that zebrafish had the highest rate of tandem (duplicates located within 10 kb of each other) and intrachromosomal (copies on the same chromosome but more than 10 kb apart from one another) duplicates of the four teleost species studied (Lu et al. 2012). Together, whole genome and local duplication events have resulted in duplicates for approximately 5300 of the 26,206 protein-coding genes identified in the zebrafish (Howe et al. 2013).

Although considered a nuisance by many (Doyle & Croll 2022; Otis et al. 2015; Vaz et al. 2019), this rich reservoir of duplicated genes offers an abundant collection of genetic data for researchers. By examining duplicated genes, it is possible to gain a greater understanding of the evolution of gene domains, the diversification of protein functions, and the complex mechanisms of gene regulation that have developed over the course of millennia. Unfortunately, researchers often focus solely on one copy of a duplicated gene, perhaps the one with the most homology to its human ortholog or the one that results in a stronger phenotype. For instance, in the case of the red-sensitive opsin genes, studies on the *lws-1* gene have been emphasized, while *lws-2* has received less attention (Xu et al. 2024). Similarly, certain transducin gene duplicates have distinct roles in vision and circadian rhythms, but those with more subtle functions linked to circadian regulation in the pineal complex are less studied compared to their counterparts involved in vision (Lagman et al. 2015). Finally, among the *cyp26* paralogs involved in retinoic acid metabolism, *cyp26a1* is more thoroughly investigated than *cyp26b1* and *cyp26c1* (Rodríguez-Marí et al. 2013). By focusing on a single paralog, we miss out on a 350-million-year natural experiment that would be difficult to reproduce in the lab.

Regardless of the disease being modeled, understanding the evolutionary history of genes duplicated in zebrafish and modeling their divergence over time is essential for creating meaningful and informative experiments. While this may require some effort, the information gleaned from these studies can inform the researcher on what insights into gene regulation and protein function can be obtained while also determining what experiments should be conducted and how their outcomes can be interpreted. Here, we will review the role of gene duplications in evolution and the existing models for gene divergence and retention . We will also demonstrate how studies that incorporate careful analyses of the post-duplication divergence of zebrafish paralogs have yielded key insights into their protein structure, function, and regulation.

## Gene duplication in evolutionary history

Gene duplication is one of the most important mechanisms driving adaptive radiation and evolutionary innovation (Arnegard et al. 2010; Crow & Wagner 2006; Kondrashov 2012; Roth et al. 2007; A. Wagner 2008). In 1970, Ohno first proposed that gene duplication is an essential mechanism driving the creation of novelty through evolution (Ohno 1970). In the context of a single-copy gene, evolutionary forces impose stringent selection pressures to maintain the integrity and functionality of the gene product. However, gene duplication results in a redundant copy, which allows for the evolution of novel functions, regulatory mechanisms, or adaptations to changing environments in the absence of stabilizing selective pressures (Force et al. 1999; Lynch & Force 2000; Magadum et al. 2013; Rochette et al. 2001). Over time, these mutations can lead to advantageous novel gene functions or increased gene specialization, leading to their long-term retention in the genome. Ohno suggested that these events occur frequently and randomly, providing a substrate for evolutionary innovation (Ohno 1970).

**Fig. 1:**
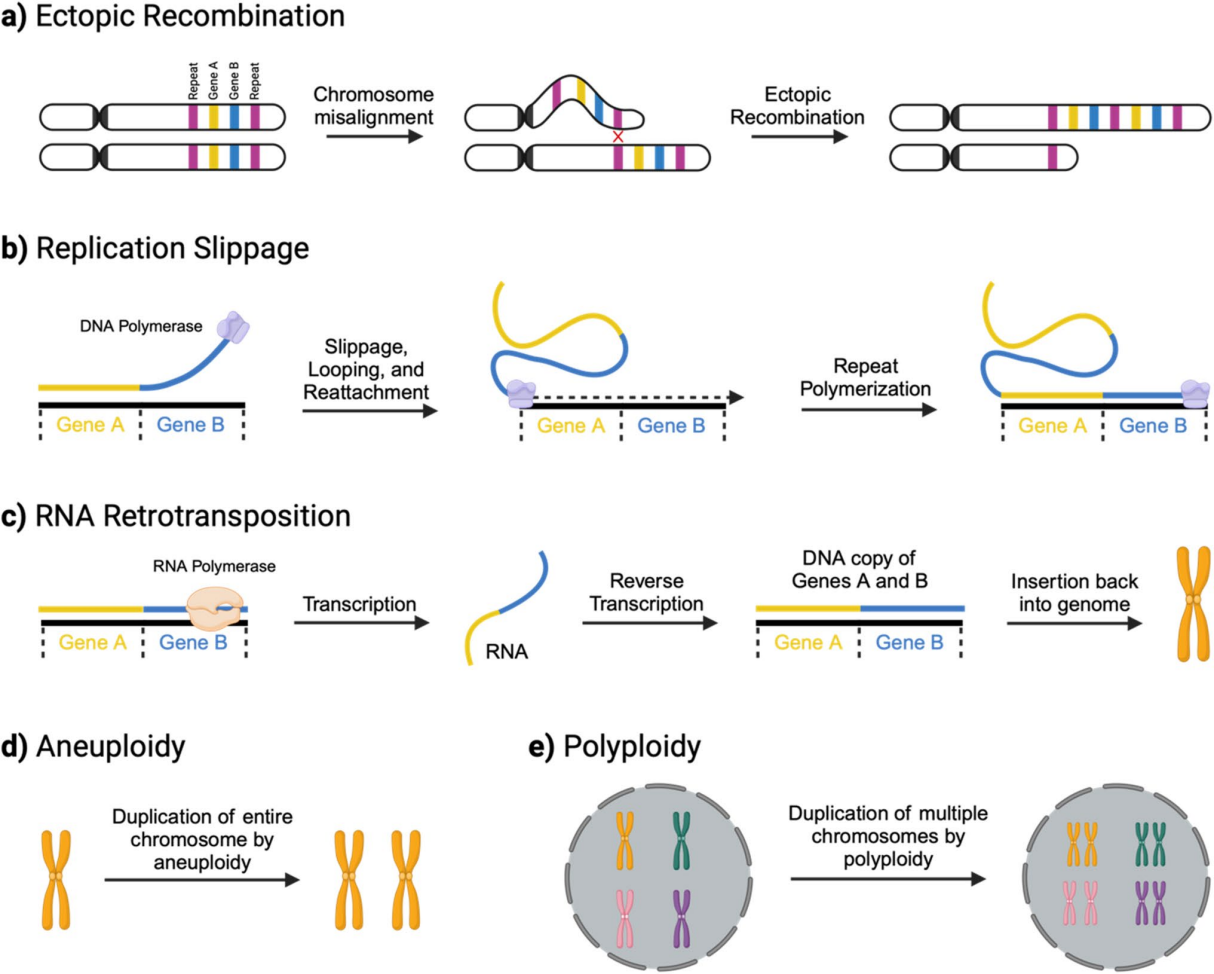
The process of gene duplication can occur through various mechanisms: **a** ectopic recombination: repeat regions (purple segments) near genes (represented by yellow and blue segments) undergo recombination following misalignment of the repeats, leading to the exchange of genetic material and the creation of duplicated genes on one of the sister chromatids. **b** Replication slippage: a DNA polymerase temporarily detaches and then reattaches to the template DNA strand. The genes will become duplicated if the DNA polymerase realigns to an area before the yellow and blue genes are located. **c** RNA retrotransposition: genes may be transcribed and then reverse-transcribed into double-stranded DNA and inserted back into the genome. **d** Aneuploidy: entire duplication of a particular chromosome due to non-disjunction errors. **e** Polyploidy: non-disjunction during meiosis results in a failure to create haploid cells, resulting in multiple copies of the genome in the offspring. Created with Biorender.com

**Fig. 2:**
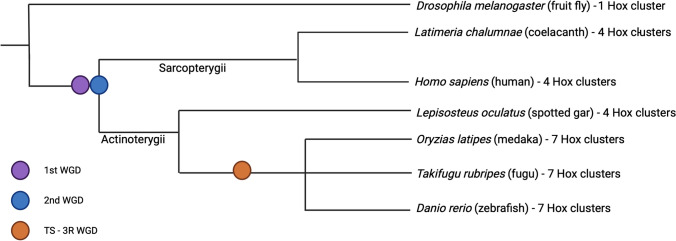
Key WGD events in vertebrate evolution. There have been at least three whole genome duplication (WGD) events in the teleost clade. The first WGD event occurred around 500 million years ago (mya) and possibly preceded the Cambrian explosion. The second WGD occurred at the base of the vertebrates or after the agnathan/gnathostome split (humans and coelacanths are shown as examples). The teleost-specific whole-genome duplication (TS-3R WGD) event occurred approximately 350 mya and led to the enormous diversity and adaptability of the teleost fish group (medaka, puffer, and zebrafish are only a few examples of this class). Created with Biorender.com

Studies across many taxa, including plants, animals, and bacteria, have corroborated Ohno’s theory that speciation events often stem from gene duplications (Anatskaya & Vinogradov 2022; Crow & Wagner 2006; Roth et al. 2007; Singh & Krumlauf 2022). For example, gene duplication is common in plants, where multiple species have undergone genome duplication several times throughout history, and many species are very tolerant of polyploidization in agricultural breeding programs (del Pozo & Ramirez-Parra 2015; Panchy et al. 2016; Qiao et al. 2019). In animals, gene duplication events are less common and usually occur on a small scale, including ectopic recombination (Christiaens et al. 2012) (Fig. 1a), replication slippage (Viguera et al. 2001) (Fig. 1b), and retrotransposition (Huang et al. 2010) (Fig. 1c)), but they can also involve larger genomic regions via aneuploidy (Koo et al. 2018) (Fig. 1d) or polyploidy (Blanc & Wolfe 2004) (Fig. 1e). The events with the most impact on evolution have been large-scale duplications, including whole genome duplication (WGD) events (Fig. 2), which have been associated with adaptive radiation in multiple clades (Meyer & Schartl 1999).

Vertebrates appear to share two rounds of WGD. The first (1R) may predate the Cambrian explosion (Meyer & Schartl 1999), and the second (2R) happened at the base of the vertebrates or after the agnathan/gnathostome split (Holland & Ocampo Daza 2018) (Fig. 2). The 1R duplication likely provided the raw genetic material that led to the development of several fundamental vertebrate features, including the complex organ systems and sophisticated neural circuitry characteristic of the phylum (Dehal & Boore 2005). The subsequent 2R duplication is believed to have resulted in further diversification of gene functions, giving rise to the vast array of vertebrate species seen today by enabling the specialization of physiological processes and morphological structures (Panopoulou & Poustka 2005). Additional WGD events have occurred more recently in vertebrate evolution, including TS-3R WGD approximately 350 million years ago, which resulted in rapid and extensive radiation of the ray-finned fishes—one of the largest vertebrate clades today.

This event is responsible for most of the genes we consider to be duplicated in the zebrafish genome. Several pieces of evidence support the TS-3R WGD (Glasauer & Neuhauss 2014). For example, many Teleostei, such as medaka, puffer fish, and zebrafish, have seven Hox clusters (Crow et al. 2006). In contrast, the Sarcopterygii, or lobed-finned fishes, and most other vertebrates have only 4 clusters of Hox genes (Ozernyuk & Schepetov 2022). There is also considerable genomic structure and comparative analysis data supporting a TS-3R WGD, including conserved synteny, zebrafish gene-mapping studies, and phylogenetic analyses of teleost genes, among others (Glasauer & Neuhauss 2014; Molinari et al. 2008; Taylor et al. 2001; Vandepoele et al. 2004).

Gene duplication, regardless of its scale or mechanism, results in one of two initial outcomes: the loss of one copy or the persistence of both. Loss of the gene, termed non-functionalization, is the most common result of gene duplication. This occurs when random mutations in key cis-regulatory or coding regions cause the gene to degenerate into a pseudogene (Evans-Yamamoto et al. 2023; Force et al. 1999; Moriyama & Koshiba-Takeuchi 2018; Rastogi & Liberles 2005). When duplicates persist, they typically exhibit one or more types of functional divergence. Subfunctionalization can split the original functions between the copies, or neofunctionalization can give rise to new functions (Birchler & Yang 2022; Force et al. 1999; Lynch & Force 2000; Qiu et al. 2020). Additionally, certain genes, like those coding for ribosomal RNA, may not undergo functional changes but are retained simply because having more copies is beneficial for the organism (Copley 2020; Hakes et al. 2007; Hallin & Landry 2019; Kuzmin et al. 2022; Xu et al. 2023). Gene families with broad expression profiles, especially those involved in developmental pathways, can also evolve after duplication to have specialized domains (Marlétaz et al. 2018). This specialization leads to more complex regulatory landscapes, particularly in neural tissues. This increase in regulatory complexity supports the idea that the retention of certain genes is advantageous because having more copies allows for greater specialization, providing significant developmental and evolutionary benefits.

Determining the evolutionary fate of gene duplicates is a dynamic interplay between hypothesis generation and experimentation. However, first determining whether a gene pair exhibits shared, split, or entirely new functions will guide what experiments to run. For instance, if both genes remain active, it might be useful to explore how their functions differ. Or, if one gene has a new role, one can look for unique traits or behaviors it supports. The experimental findings can then confirm or refute the initial hypotheses about the duplicated genes.

The study of the *elastin b* (*elnb*) gene in zebrafish provides a clear illustration of this principle (Moriyama et al. 2016). Researchers discovered that *elnb*, which was created during the TS-3R WGD, neofunctionalized and became essential for the proper development of the bulbus arteriosus (BA) by regulating the differentiation of cardiac precursor cells into smooth muscle. Rescue experiments with *elnb* mRNA mitigated the knockdown phenotype. However, when attempting to rescue the *elnb* morphant phenotype with the ancestral *elastin* gene from *Polypterus senegalus*, a basal actinopterygian and non-teleost fish considered to possess an ancestral-like gene due to its evolutionary position, the experiments did not yield the same restorative effects, implying a novel function for the duplicated *elnb* gene in zebrafish BA development. This process elucidates the creation of an experimental feedback loop where initial experimental data shapes subsequent hypotheses and directs future research, deepening our comprehension of how genetic evolution influences an organism’s adaptability and development. Below, we will examine examples of studies conducted on genes exhibiting each of the major post-duplication models and the insights gained from each (Table 1). These selected studies highlight key aspects of gene duplication research, demonstrating how such studies contribute to our understanding of genetic diversity and functionality across species.

**Table 1:** Comparative analysis of duplicate genes. Similarity, expression patterns, and functional divergence

Duplicate genes	Human ortholog	Amino acid similarity	Overlapping expression	Differential tissue distribution	Origin of the paralogs	Functional divergence	Compensatory expression	Reference
*elna* and *elnb*	*ELN*	–	Y	Y	TS-3R WGD	Y	N	(Moriyama et al. 2016)
*Sox9a* and *sox9b*	*SOX9*	–	Y	Y	TS-3R WGD	Y	N	(Yan et al. 2005)
*oxr1a* and *oxr1b*	*OXR1*	86%	Y	Y	Segmental sequence duplication	Y	N	(Xu et al. 2020, 2021)
*Pax6a* and *pax6b*	*PAX6*	95%	Y	Y	TS-3R WGD	Y	N	(Kleinjan et al. 2008; Thummel et al. 2010)
*nrf2a* and *nrf2b*	*NRF2*	25%	Y	Y	TS-3R WGD	Y	N	(Sant et al. 2017; Timme-Laragy et al. 2012)
*tbx5a* and *tbx5b*	*TBX5*	83%	Y	N	TS-3R WGD	Y	N	(Anderson et al. 2022; Parrie et al. 2013)
*znf143a* and *znf143b*	*ZNF143*	65%	Y	N	TS-3R WGD	N	Y	(Huning & Kunkel 2020)
*Scn1laa* and* scn1lab*	SCN1A	67%	N	Y	–	Y	N	(Weuring et al. 2022)
*igf1a*, *igf1b*, and *igf2a*, *igf2b*	*IGF-1* and *IGF-2*	50% and 70%	Y	Y	TS-3R WGD	Y	N	(Zou et al. 2009)
*hoxa13a* and *hoxa13b*	*HOXA13*	–	Y	Y	TS-3R WGD	Y	–	(Crow et al. 2009)
otos and otospiralin like	*OTOS*	28%	Y	Y	–	Y	N	(Baanannou et al. 2020)
*dscama* and *dscamb*	DSCAM	83%	Y	Y	TS-3R WGD	–	–	(Galicia et al. 2018)
*rbp7a* and *rbp7b*	*RBP7*	80%	Y	Y	TS-3R WGD	–	–	(Belliveau et al. 2010)
*foxl2a* and *foxl2b*	*FOXL2*	64%	Y	Y	–	Y	–	(Yang et al. 2017)
*en2a* and *en2b*	*EN2*	79%	Y	Y	TS-3R WGD	Y	–	(Scholpp & Brand 2001)
*rh1* and *rh1-20/0/0000 0:00:00 AM*	*RH1*	75%	Y	Y	Zebrafish-specific duplication	–	–	(Morrow et al. 2011)
*dmrt2a* and *dmrt2b*	*DMRT2*	–	Y	Y	TS-3R WGD	Y	–	(Liu et al. 2009)
*atf5a* and *atf5b*	*AFT5*	40%	Y	Y	TS-3R WGD	–	N	(Rodríguez-Morales et al. 2020; Zhu et al. 2022)
*igfbp-1a* and *igfbp-1b*	*IGFBP1*	50%	Y	Y	TS-3R WGD	N	–	(Kamei et al. 2008)
*cryaa* and *cryabb*	*CRYAB*	50%	N	Y	TS-3R WGD	Y	–	(Smith et al. 2006)
*crabp1a* and *crabp1b*	*CRABP1*	88%	N	Y	TS-3R WGD	N	–	(R.-Z. Liu et al. 2005)
*atxn1a* and *atxn1b*	*ATXN1*	35%	Y	Y	TS-3R WGD	–	–	(Vauti et al. 2021)
*dmbx1a* and *dmbx1b*	*DMBX1*	72%	Y	Y	TS-3R WGD	Y	N	(Chang et al. 2006; Wong et al. 2010)
*inpp5ka* and *inpp5kb*	*INPP5K*	56%	Y	Y	TS-3R WGD	Y	–	(Shukla et al. 2022)
*stat5.1* and *stat5.2*	*STAT5*	–	Y	–	Zebrafish specific duplication	N	N	(Lewis & Ward 2004)
*sox11a* and *sox11b*	*SOX11*	75%	Y	Y	WGD	N	N	(de Martino et al. 2000)
*mdka* and *mdkb*	*MDK*	72%	Y	Y	Segmental sequence duplication	Y	–	(Winkler et al. 2003)

A detailed comparison of various duplicated genes in relation to their human orthologs, amino acid similarity, expression patterns, tissue distribution, origin, functional divergence, compensatory expression, and literature references. All genes included in this table are derived from studies conducted on zebrafish. **Duplicate genes**: list pairs or groups of duplicated genes studied. **Human ortholog**: the corresponding human gene equivalent, if applicable. **Amino acid similarity**: percentage similarity between the amino acids of the duplicated genes in zebrafish compared to each other. **Expression**: indicates whether the genes share expression patterns in the same tissues. **Differential tissue distribution**: indicates if the genes are expressed in different tissues. **Origin of the paralogs**: describes the evolutionary origin of the gene duplication, such as the teleost-specific third round whole genome duplication (TS-3R WGD) or segmental sequence duplication (SSD). **Functional divergence**: indicates whether the duplicated genes have evolved to perform different functions post-duplication. **Compensatory expression**: indicates whether one gene compensates for the loss or reduction in function of its duplicate. **Reference**: citations of the studies or reviews from which the data were derived. A dash (“–”) indicates that this information was not provided.

*Y* yes, *N* no.

## Models for post-duplication evolution

### Non-functionalization

**Fig. 3:**
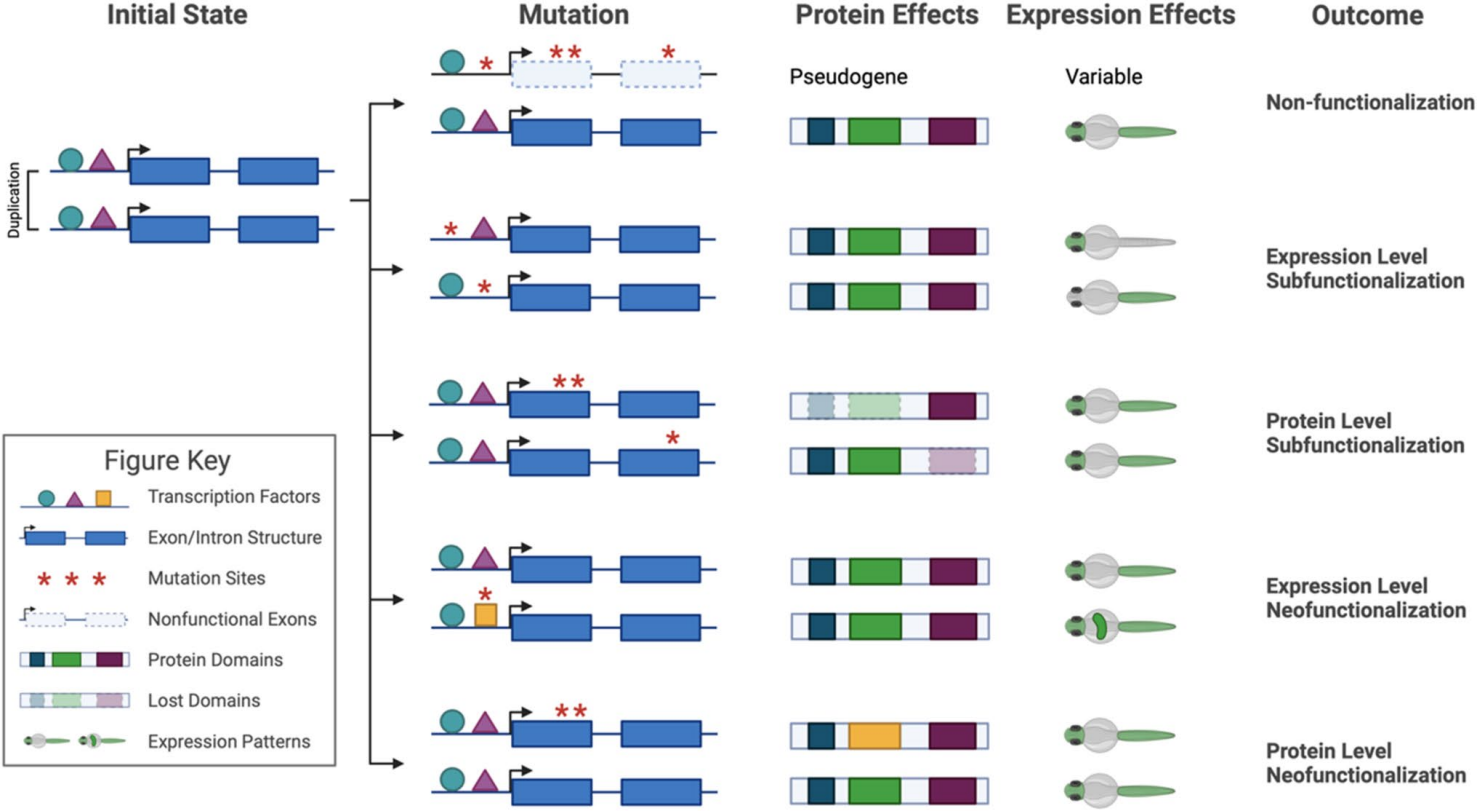
Schematic representation of the different fates of duplications in zebrafish. Example protein domains are shown in blue, green, and dark purple. Hypothetical regulatory proteins are shown as a blue circle, purple triangle or yellow square. 1st outcome—non-functionalization: One of the gene copies acquires a degenerative mutation that turns it into a pseudogene (shown in gray boxes). This gene is no longer used, and only one functional copy remains. 2nd outcome—expression level subfunctionalization: Differential regulation leads to each gene copy to be expressed in specific patterns, allowing distinct but complementary functionalities. 3rd outcome—protein level subfunctionalization: Mutations modify protein domains, changing their functions and allowing each gene copy to fulfill subsets of the original function**.** 4th outcome—expression level neofunctionalization: Novel expression patterns emerge in one of the gene copies, providing new functional capabilities. 5th outcome—protein level subfunctionalization: Unique mutations in one gene copy confer an entirely new protein function, enhancing biological diversity and adaptability. Created with Biorender.com

The most common outcome following a gene duplication event is the formation of a pseudogene. While duplication relaxes selective pressure and allows for the rapid exploration of the evolutionary space, this often simply leads to the accumulation of deleterious mutations—leading to a loss of gene function (Fig. 3: non-functionalization). Pseudogenes are sequences of DNA that resemble functional genes but have lost their gene expression or protein-coding ability due to these mutations. In many cases, this results in a return to the ancestral state, but non-functionalization can also lead to novel traits. The teleost globin superfamily is a striking depiction of the evolutionary innovation from non-functionalization (Hoffmann et al. 2021). Multiple functional globin isoforms have accommodated unique environmental and developmental challenges in many niches among the teleost species (Storz et al. 2020). These specialized isoforms appear to have arisen from repeated rounds of tandem duplication, independent evolution of the two copies, and non-functionalization of the less fit duplicate (Opazo et al. 2013; Storz et al. 2013; Tiedke et al. 2011). For example, the η-globin gene is a pseudogene in all primates. This classification is based on sequence analyses that show accumulated mutations like frameshifts and premature stop codons, which disrupt the gene’s ability to produce a functional protein. Similarly, the δ-globin gene is often found to be a pseudogene in many eutherian species due to gene conversion events where sequences from the β-globin gene are copied into the δ-globin gene locus (Hardison 2012). Although these conversions result in non-functional genes, the evolutionary interval where duplicated genes retain their original function before any significant divergence or loss occurs, referred to as lag, allows for the duplicated genes to be subjected to genetic drift and varying selective pressures (Lynch & Conery 2000). Thus, due to the lag between duplication and non-functionalization, traits can evolve under conditions with lower selective pressure and then be tested for fitness via the non-functionalization of one of the duplicates (Conant & Wolfe 2008; Innan & Kondrashov 2010; Zhang 2003).

Even when pseudogenization occurs, these former duplicated copies are worth exploring. Once thought to be molecular “fossils” with no function, pseudogenes play a role in many cellular processes by expressing small interfering RNA, competitive endogenous RNA, or antisense transcripts (Groen et al. 2014). Thus, they are involved in complex genetic regulation both at the transcriptional and post-transcriptional levels. For example, they play a critical role in cancer progression, particularly in kidney cancer, and display tissue-specific expression patterns, indicating unique regulatory roles in different tissues (Nakamura-García & Espinal-Enríquez 2023; Qian et al. 2022; Welch et al. 2015). Disease-related changes in cellular processes can also lead to the reactivation or altered expression of pseudogenes (Pink et al. 2011), providing the potential for disease subtyping and personalized treatment decisions (Chen et al., 2020). Specific pseudogene expression changes are often highly specific to certain diseases or disease subtypes, contributing to high diagnostic accuracy and predictive power (Glenfield & McLysaght 2018; Y. Ma et al. 2021; Roberts & Morris 2013).

One key aspect to consider when studying pseudogenes is their cis-regulatory elements. Highly conserved cis-regulatory modules (CRMs) tend to be preserved alongside gene copies that retain their functional roles. In contrast, when a gene duplication results in a non-functional copy or a pseudogene, we often see a concomitant degradation of associated CRMs. Thus, the presence and conservation of CRMs in the vicinity of pseudogenes may indicate that these genetic elements are actively involved in regulating nearby functional genes within the same regulatory network (Kikuta et al. 2007). This suggests that the pseudogenes themselves may not be the direct targets but rather bystanders, with the CRMs playing a crucial role in the regulation of other genes. On the other hand, degradation of CRMs suggests a diminished role in the organism’s current genetic regulation and evolutionary progression.

### Subfunctionalization

Subfunctionalization, where the ancestral gene expression pattern or domain functions are partitioned between the duplicates, is the most common fate that results in the long-term preservation of functional paralogs in eukaryotic genomes (Lynch & Force 2000). Several cases in zebrafish have been reported where each paralog in a duplicate gene pair performs a subset of the functions performed by a single gene in tetrapods.

The most well-studied mechanism for this division of labor is expression pattern divergence (Fig. 3: expression level subfunctionalization). This commonly occurs by changing spatial expression domains, exemplified by *OXR1* (oxidation resistance gene 1) (*oxr1a* and *oxr1b* in zebrafish) and *PAX6* (Paired box protein Pax-6) (*pax6a* and *pax6b* in zebrafish). In mammals, *OXR1* is highly expressed in the brain, particularly in regions such as the cerebral cortex, hippocampus, and cerebellum, reflecting its role in neuroprotection. It is also expressed in other tissues, including the heart, liver, and kidney, which aligns with its functions in antioxidant defenses and cellular stress responses (Oliver et al. 2011; Volkert & Crowley 2020). In zebrafish, *oxr1a* is maternally expressed and ubiquitous from the two-cell to the sphere stage, later localizing to the head, including the brain, olfactory bulbs, retina, and neurons by 30 h post-fertilization (hpf). In adulthood, *oxr1a* is highly expressed in the brain, eye, and testis, indicating roles in development, reproduction, and antioxidant defenses. *oxr1b* is strongly expressed during the cleavage period and is restricted to the head, specifically in the lateral line ganglia and spinal cord neurons during the pharyngula period (24–48 hpf). By 48–72 hpf, *oxr1b* is also present in the olfactory bulbs, eye, adenohypophysis, and neuromasts. In adulthood, *oxr1b* has higher expression levels compared to *oxr1a* in most tissues. Although both genes are expressed in the brain, olfactory bulbs, and eye, they exhibit distinct spatial domains: *oxr1a* is prominent in the retina and heart, while *oxr1b* is found in the adenohypophysis and neuromasts (Xu et al. 2020, 2021). These patterns indicate subfunctionalization in the zebrafish model, reflecting a division of the expression domain of human.

Another well-studied pair, *pax6a* and *pax6b*, is a noteworthy example of spatial subfunctionalization in the brain and pancreas. Studying the non-coding regions that control them has led to the identification of distinct enhancer elements that regulate different aspects of Pax6 expression (Delporte et al. 2008; Kleinjan et al. 2008; Thummel et al. 2010). Enhancers that drive expression in the pancreas versus the brain are different, and these can be pinpointed by looking at where and when each paralog is expressed and then comparing their respective cis-regulatory elements with pre-duplication outgroups. This knowledge can inform the understanding of human diseases related to *PAX6*, such as diabetes mellitus, which affects the pancreas, and aniridia, which presents with neurological issues (Gosmain et al. 2012; Landsend et al. 2021; Lim et al. 2017; Moosajee et al. 1993; Sekiya et al. 2022; Uttley et al. 2023). Furthermore, it may provide novel insights into the development of therapeutic approaches that target these enhancers to modulate *PAX6* expression in these and other disease conditions.

In addition to spatial divisions of labor, expression-based subfunctionalization can also act on temporal expression patterns. One such example is *NRF2* (nuclear factor erythroid 2-related factor 2). The distinct temporal expression patterns of the *nrf2a* and *nrf2b* paralogs are a critical factor in the zebrafish’s response to oxidative stress. Morpholino-induced knockdown experiments showed that while both *nrf2a* and *nrf2b* influence the glutathione redox state, their impact is not uniform throughout development. Notably, *nrf2a* morphants exhibited an oxidized glutathione redox potential and altered glutathione dynamics starting at 48 hpf, indicative of *nrf2a*’s active role during mid-embryogenesis in regulating the oxidative stress response. In contrast, *nrf2b* knockdown led to a reduction in the glutathione redox potential at 3 hpf, with later developmental stages resembling control embryos (Sant et al. 2017; Timme-Laragy et al. 2012).

Not all examples of subfunctionalization involve discernable differences in expression patterns. Subfunctionalization can result from protein domain modulation, where the duplicates divide the original function at the coding sequence level (Fig. 3: Protein level subfunctionalization). As a result, neither of the duplicates retains the complete set of functions of the original protein. This type of subfunctionalization has largely been dismissed as a rare exception, but gene expression analysis using modern transcriptomics techniques has shown that it may be more common than previously thought. For example, in a study by Hill et al., (2017), gene expression analysis in the zebrafish heart revealed that over 10% of duplicated genes retained similar temporal and spatial expression patterns.

Despite being assumed to be rare, a few examples of studies on protein-level subfunctionalization can be found. One well-characterized example of protein-level subfunctionalization was provided by Parrie et al. (2013), who demonstrated, despite co-expression in developing heart and limb, Tbx5a and Tbx5b display distinct amino acid sequences that confer unique functions. The T-box domain of Tbx5b shares only 83% sequence identity with its Tbx5a counterpart, which is significantly lower than the typical 95–99% sequence identity observed between paralogous T-box genes within the same subfamily. This divergence is reflected in their independent essential requirements for cardiac and fin development, as injection of mRNA from one *tbx5* paralog was unable to compensate for the loss of the other (Parrie et al. 2013). This domain-specific understanding is helpful because it allows for a granular view of protein functionality, which is essential for designing targeted therapies that can modulate specific protein activities without affecting others. Additionally, this knowledge can be pivotal in understanding the molecular basis of diseases caused by mutations in specific protein domains of TBX5, paving the way for precision medicine approaches and improved genetic diagnostics.

In many cases, duplicated genes can be retained without discernable differences in expression pattern or gene function. This can sometimes be explained by changes in gene expression levels where each paralog is expressed at lower levels than the ancestral gene, thus requiring two copies to maintain sufficient protein levels in the cell. In other cases, an increase in the gene expression, or dosage, benefits the cell, leading to increased fitness. A study by Ihmels et al. (2007) observed that in yeast, certain duplicate genes, particularly those involved in dosage amplification like histone genes, maintain high and correlated expression levels post-duplication. This correlation in expression patterns is indicative of a co-regulation mechanism, suggesting that these gene duplicates are preserved at high abundance to meet cellular demands. Thus, cells may derive a selective advantage from such duplication due to the increased dosage of critical gene products, although the overall impact of duplicates on genetic robustness appears to be modest.

Studying subfunctionalization can provide insights into the dosage sensitivity of their human orthologs, especially those that exhibit haploinsufficiency phenotypes. Haploinsufficiency occurs when a single copy of a gene is not sufficient to maintain normal function. Although clear examples of haploinsufficient genes in humans being studied in zebrafish have been published, expression modulation to compensate for the loss of a duplicate is a well-characterized phenomenon in zebrafish (El-Brolosy et al. 2019). For example, Zinc Finger Protein 143 (ZNF143) is a sequence-specific transcriptional activator that plays a critical role in the regulation of both mRNA and small nuclear RNA gene promoters. In a study by Huning and Kunkel (2020), the knockout effects of the two paralogous genes, *znf143a* and *znf143b*, in zebrafish were investigated using CRISPR interference (CRISPRi). The study used in situ hybridization to analyze the expression of these two genes at 24 h post-fertilization (hpf). The results showed that both *znf143a* and *znf143b* mRNAs are strongly expressed in the brain regions, including the forebrain, midbrain, and hindbrain. Despite their similar spatial expression, there is a notable difference in their expression levels during early development. The knockdown results showed that knocking down either *znf143a* or *znf143b* led to similar phenotypic defects in zebrafish embryos. They also observed that knocking down *znf143a* led to a 1.5-fold increase in *znf143b* mRNA levels, indicating a compensatory mechanism that attempts to maintain functional Znf143 protein levels. This compensatory response was not reciprocated when *znf143b* was knocked down. The study concludes that the tight control of gene dosage is likely essential for maintaining developmental processes and that disruptions in this balance can lead to significant morphological and functional defects. Abnormal expression levels of *znf143*, either too high or too low, could potentially lead to similar disruptions as seen in haploinsufficiency, affecting cell cycle regulation. In the case of WGD, organisms might develop mechanisms to balance the expression levels of all duplicated genes to prevent deleterious effects of over- or under-expression. On the other hand, tandem duplications, which occur when genes are duplicated within the same chromosome, often lead to dosage imbalances. This imbalance can be detrimental if the duplicated gene is dose-sensitive, as is the case with many genes involved in genetic disorders (Birchler & Yang 2022; Rice & McLysaght 2017). To summarize, the impact of dosage imbalance due to duplication can be either advantageous or detrimental, depending on the specific gene involved.

Similar research can also elucidate specific aspects of gene function that might be obscured in species where these functions are still combined in a single gene. For example, Dravet syndrome in humans is caused by heterozygous loss-of-function mutations in the *SCN1A* (sodium voltage-gated channel alpha subunit 1) gene. This protein is pivotal for initiating and propagating action potentials in neurons, with a pronounced expression in inhibitory interneurons of the central nervous system. The dysfunction of these channels, due to the mutations, compromises neuronal inhibition, leading to the neuronal hyperexcitability observed in seizure disorders (Martins Custodio et al. 2023). The phenotypic spectrum of Dravet syndrome encompasses seizures (generalized, focal, or unilateral), cognitive impairments (visual troubles, delayed motor skills, speech, and attention), and movement disorders (ataxia, choreoathetosis, and gait issues). This phenotypic spectrum is influenced by several factors related to the *SCN1A* gene mutations. The class of variant—whether missense, nonsense, or frameshift—has distinct effects on the protein function (Chen et al. 2022, b; Gonsales et al. 2019; Ma et al. 2022). Missense mutations may partially alter channel function, while nonsense or frameshift mutations could result in a complete loss of function. Although Dravet syndrome has been extensively studied in humans, the full phenotypic spectrum and the nuances of genotype–phenotype correlations are not yet fully understood.

Dravet syndrome represents a clear use-case where duplications in zebrafish present a valuable model for study. The zebrafish paralogs *scn1laa* and *scn1lab* not only have different spatial expression patterns but also exhibit distinct phenotypic traits (Weuring et al. 2022). Significant attention has been directed towards the *scn1lab* gene in zebrafish, as it is the more conserved ortholog of the human *SCN1A* gene, and mutations in *scn1lab* have been consistently linked to epilepsy phenotypes in zebrafish, echoing the pathological features observed in Dravet syndrome (Griffin et al. 2017; Schoonheim et al. 2010; Sourbron et al. 2016; Weuring et al. 2020). However, the *scn1laa* gene, despite being less studied, has been shown to influence neuronal excitability as well. Mutant phenotypes of *scn1laa* under standard conditions present with altered brain and forebrain transmission of nerve impulses, supporting *scn1Laa*’s involvement in the neurological pathways relevant to Dravet syndrome (Griffin et al. 2017; Weuring et al. 2022).

The idea that research should focus solely on *scn1lab*, as suggested by some in the field (Weuring et al. 2022), overlooks the complexity of genetic interactions and the potential insights offered by studying both paralogs. In zebrafish, *scn1laa* and *scn1lab* likely have undergone subfunctionalization or neofunctionalization as mutants display partially overlapping phenotypes. By studying both paralogs, we can uncover potential compensatory mechanisms that one gene may exert in the presence of mutations in the other. Such compensatory interactions can have critical implications for understanding the variability and penetrance of *SCN1A*-related disorders. Exploring differential drug responses that might not be apparent when examining a single paralog may also pave the way for more personalized approaches to treatment.

Another example is Insulin-like Growth Factor (IGF). Zebrafish possess four distinct IGF genes: *igf-1a*, *igf-1b*, *igf-2a*, and *igf-2b* (Zou et al. 2009). This duplication has permitted the subfunctionalization of IGF proteins, with each evolving distinct expression patterns and physiological roles. In zebrafish, the IGF genes *igf*-*2a* and *igf-2b* exhibit high sequence identity with human *IGF-2* and with each other. However, *igf-2b* mRNA is specifically expressed in the liver, while *igf-2a* mRNA is widely expressed across tissues, likely reflecting the partitioning of function among these genes. The presence of two distinct 5′-UTR sequences in both zebrafish *igf-2a* and *igf-1b*, resulting in different transcription initiation sites and signal peptides, further underscores the evolutionary pressure to maintain distinct physiological roles for these paralogs (Zou et al. 2009). These divergent expression profiles suggest that studying these genes in zebrafish can provide deeper insight into their individual contributions to tissue development and homeostasis. In humans, dysregulation of IGF signaling is implicated in diseases ranging from growth disorders to cancer (Murrell et al. 2004). By dissecting the distinct roles of *igf-2a* and *igf-2b* in zebrafish, we can gain valuable insights into the tissue-specific functions and regulatory mechanisms of IGF signaling, potentially leading to targeted therapies in human diseases where IGF is dysregulated.

In summary, the study of subfunctionalized genes in zebrafish enhances our understanding of gene function by allowing us to see how individual aspects of a gene’s role evolve and operate in a biological context. This information can be crucial for understanding similar processes in humans, where such functional distinctions might not be as easily observable.

### Neofunctionalization

Neofunctionalization is the process through which functional divergence occurs when one of the duplicated genes obtains a novel function advantageous to the organism, and the gene is retained (Hurles 2004) (Fig. 3: neofunctionalization). It is difficult to study due to the low probability of a gene acquiring a new function, leading to a natural paucity of cases. In addition, pinpointing the original gene function is challenging, making it hard to prove changes in protein function. However, recent advances in computational methods for ancestral gene reconstruction and protein modeling combined with the rapid proliferation of reference genomes may make neofunctionalization easier to identify across evolutionary timelines (Cai et al. 2004; Finnigan et al. 2012; Joy et al. 2016; Nocedal & Laub 2022; Ogawa et al. 2013; Scossa & Fernie 2021; Voordeckers et al. 2012). Neofunctionalization typically co-occurs with subfunctionalization, with one gene adopting new roles while preserving a subset of ancestral functions.

Although rare, a few confirmed cases of neofunctionalization exist in the literature. One well-documented instance of this is observed in the gene duplication of *hoxa13a* and *hoxa13b* in zebrafish. The gene *hoxa13a* exhibits hypermutability, which has allowed it to accumulate a higher number of mutations when compared to its paralog *hoxa13b*. Such hypermutability has been linked to the development of the median fin fold (MFF) in zebrafish, a specialized fin structure observed in the early development of many teleost fish. This suggests that the function associated with *hoxa13a* and its hypermutability is a novel role rather than one retained from the ancestral gene function before duplication (Crow et al. 2009). This type of mutation-driven divergence is crucial in the evolution of species as it provides the genetic variability on which natural selection can act, giving rise to new phenotypes that can be subject to evolutionary pressures.

### Complex evolutionary fates

In many cases, two or more of the evolutionary fates discussed above happen together. For example, duplicates may exhibit a combination of the expression pattern and protein modulation forms of subfunctionalization. In other instances, duplicate genes have similar and unique functions that are required in tandem with one another but at different times in development. Dosage compensation is often accompanied by various forms of subfunctionalization (Hultman et al. 2007; Lagman et al. 2015; Leerberg et al. 2019; Sedletcaia & Evans 2011). For example, *hoxb1a* and *hoxb1b* in zebrafish have significant functional redundancy as each of them is capable of promoting Mauthner neuron differentiation and rescuing the defects caused by knockdown of *hoxb1b* while also performing unique functions by themselves (McClintock et al. 2002). In this particular situation, the “Piggyback hypothesis,” which posits that the preservation of redundant parts of the gene is influenced by structural constraints within the genome, provides further insight (Qian et al. 2010). This preservation is thought to be influenced by the proximity of the gene segment that codes for a unique function to the redundant part, which is why the redundant function remains in the genome. Other combinations where the duplicated genes both subfunctionalize and neofunctionalize are possible, although cases have only recently begun to be identified.

## Impact on human health

Comparative studies that analyze and compare both duplicate genes in zebrafish can inform research into human biology in at least four important ways. First, comparisons of the structural and functional differences between pairs can be used to identify the roles of specific domains and enhancers. Paralogs can evolve to possess distinct cis-regulatory elements that drive their expression in different tissues or developmental stages, leading to functional diversification. For instance, the zebrafish *sox9a* and *sox9b* genes, which arose from the teleost-specific genome duplication, have been shown to possess distinct functions in craniofacial and pectoral fin development due to their distinct expression patterns; *sox9a* is expressed predominantly in the somites and pharyngeal arches while *sox9b* is more prominent in the eye and otic vesicle (ear) (Yan et al. 2005). Consistently, loss of *sox9b* leads to ear defects and reduced craniofacial cartilage. Conversely, the lack of *sox9a* affects chondrocyte stacking in cartilage, which could be due to alterations in domains responsible for chondrogenic differentiation. Thus, the specific functions of *sox9* in each of these tissues can be more easily parsed out in the zebrafish than it can in mammalian models where a single gene is involved in both processes.

The second way that duplicate gene studies can inform our understanding of human biology is by comparative analysis of gene clusters. For instance, the Hox gene clusters, which are fundamental for the development of the body plan, have been refined through multiple whole-genome duplication events. In early vertebrates, two rounds of genome duplication events (2R) produced the four paralogous Hox clusters (HoxA, HoxB, HoxC, and HoxD) (Fig. 2) widely conserved across most species (Ozernyuk & Schepetov 2022; Singh & Krumlauf 2022; Soshnikova et al. 2013; G. P. Wagner et al. 2003). Teleosts, however, have seven or eight Hox clusters due to the TS-3R WGD and subsequent gene loss in some species (Amores et al. 1998; Málaga-Trillo & Meyer 2001; Pascual-Anaya et al. 2013; Yamada et al. 2021). By comparing the differences and similarities in how these genes function in zebrafish and humans, researchers can gain insights into their role in development and disease. For example, Dietrich et al. (2021) compared Hox gene expression patterns between zebrafish in the developing limb and highlighted how both the similarities and differences between genes and expression patterns can be used to model human skeletal diseases like osteogenesis imperfecta and osteopetrosis. This comparative genetic approach underscores the importance of *Hox* genes in vertebrate skeletal formation, disease phenotypes and potential therapies.

Another example of gene cluster evolution is the duplication of the Major Histocompatibility Complex (MHC) genes, which are crucial for the immune response to pathogens (Cruz-Tapias et al. 2013). *MHC* genes have undergone several rounds of duplication in both humans and zebrafish (Sambrook et al. 2005). In humans, *MHC* gene expansion is primarily due to tandem duplications (Traherne 2008). Zebrafish also share this mechanism, but uniquely, they have further diversified their MHC genes through the TS-3R WGD (Bingulac-Popovic et al. 1997; Dirscherl et al. 2014). This has led to a wide variety of MHC class I genes in zebrafish, categorized into U, Z, and L lineages, dispersed across multiple chromosomes (Dirscherl et al. 2014). By comparing the evolution of the more extensive and more complex MHC repertoire in zebrafish, we can uncover how the interplay between unique immunological threats and gene duplication have driven the evolution of our immune system (Bingulac-Popovic et al. 1997; Dirscherl et al. 2014).

Third, gene duplication in zebrafish can also serve as a model for human diseases caused by gene duplication or amplification. For instance, gene duplication events are associated with certain cancers in humans where the extra copies of genes lead to overexpression and tumorigenesis (Baines et al. 2022; Glenfield & Innan 2021). A prime example is the amplification of the *HER2* gene, which occurs in about 15–20% of breast cancers. Increased copy numbers of *HER2* result in continuous growth signals that contribute to the uncontrolled proliferation characteristic of cancer. In this context, zebrafish offer a powerful model for studying the implications of gene duplication, as *her2* is duplicated in the zebrafish genome but without the typical oncogenic effects (Cappuzzo et al. 2005; Chen et al. 2022, b).

Finally, studying gene duplication can also contribute to understanding chemotherapy resistance, as duplication in cancer cells can result in overexpression and, consequently, the development of resistance to certain drugs (Glenfield & Innan 2021; Wu et al. 2015). An early illustration of the critical impact of gene duplication in cancer resistance was the discovery of the dihydrofolate reductase (*DHFR*) gene’s duplication in 1978 (Alt et al. 1978). The DHFR enzyme, which is targeted by the drug methotrexate, is vital for DNA synthesis and cellular proliferation (Askari & Krajinovic 2010). However, duplication of the DHFR gene results in increased enzyme production, enabling cancer cells to overcome the effects of methotrexate (Alt et al. 1978; Glenfield & Innan 2021; Turner et al. 2017). Future studies on the duplication and diversification of these and other genes involved in cancer will increase our understanding of how gene overexpression following duplication promotes oncogenesis, potentially paving the way for novel therapeutic approaches in treating human cancers (Kalkat et al. 2017).

## Conclusion

Studying how genes duplicate and evolve is crucial for understanding their structure, function, and regulation and how changes in these factors can lead to evolution and disease. Post-duplication, these genes acquire mutations more freely, fostering new functions and aiding adaptation. Regardless of whether duplicated genes remain unaltered, become pseudogenes, or evolve novel functions, examining each outcome can shed light on evolutionary innovations and identify factors for genetic disease modeling and therapeutic development. In addition, analysis of pseudogene formation provides new perspectives on gene expression regulation and genome stability relevant to cancer genomics and the identification of genetic disease markers.

However, in zebrafish research, certain paralogous genes have generally been studied more extensively compared to their paralogs. This pattern demonstrates the tendency in genetic research to concentrate on genes that exhibit more pronounced or observable phenotypic effects or are more closely related to their human counterparts, potentially overshadowing the significant roles of their paralogs. In such cases, valuable information about how evolution drives functional innovation and how that innovation may affect disease and even potential treatments may be missed. More importantly, the results from genetic manipulations of one paralog in zebrafish may not accurately represent the full repertoire of gene functions for its ortholog in humans, as gene expression patterns and protein domain functions may be partitioned between the two paralogs. These factors cannot be ignored if we want to maximize the utility of zebrafish as a model for human disease.

A well-designed study for the duplicates should include at least two components: (1) Identification of the expression domain of the paralogs and (2) functional analysis, such as generating single and double knockouts, to identify individual functions and check for redundancy. Additional supportive information can be obtained through rescue experiments to check for compensation, analysis of the phylogenetic relationship with the ancestral gene, and examination of expression patterns across different developmental stages. However, employing heterologous expression systems comes with challenges, such as potential discrepancies in post-translational modifications, differences in cellular environments, and the intricacies of gene regulation across species. Despite these obstacles, the strategic use of domain deletions or switches between duplicated genes in such systems can elucidate protein domains’ roles in mediating specific processes and phenotypes, thereby confirming the molecular basis for particular functions.

Although the specific applications of these methods will vary on a case-by-case basis, the key is that both paralogs are studied together. Only by comparing paralogs can we understand how evolutionary pressures shape gene expression and protein function. Only by comparing paralogs can we accurately model human disease in the zebrafish. And only by expanding our view to embrace the unique information embedded in the zebrafish genome can we fully utilize this vast genetic resource to advance our understanding of nature and disease.

## Data Availability

No datasets were generated or analysed during the current study.
